# Prevalence of lung lesions in slaughtered cattle in the Eastern Cape Province, South Africa

**DOI:** 10.4102/jsava.v87i1.1362

**Published:** 2016-10-26

**Authors:** Ishmael F. Jaja, Borden Mushonga, Ezekiel Green, Voster Muchenje

**Affiliations:** 1 Department of Livestock and Pasture Science, University of Fort Hare, South Africa; 2 Faculty of Agriculture and Natural Resources, University of Namibia, Namibia; 3 Department of Biochemistry and Microbiology, University of Fort Hare, South Africa

## Abstract

Information obtained from abattoirs on the causes of lung condemnation is important in preventing the spread of zoonotic diseases and for promoting food security. In this study, we assessed the causes of lung condemnation in cattle at three abattoirs represented as ANA, QTA and EBA to evaluate the financial losses associated with lung condemnation. A retrospective study (*n* = 51 302) involving the use of abattoir slaughter records of 2010–2012 and an active abattoir survey (*n* = 1374) was conducted from July to December 2013. The retrospective study revealed the main causes of lung condemnation as pneumonia (1.09%, 2.21% and 0.77%), emphysema (1.12%, 1.14% and 1.1.6%) and abscessation (0.71%, 1.06% and 0.77%), from ANA, QTA and EBA, respectively. The combined monetary loss because of lung condemnation during the period 2010 to 2012 was estimated as ZAR 85 158 (USD 7939) for the abattoirs surveyed. Conversely, during the active abattoir survey, agonal emphysema (15%, 15% and 23%) and improper eviscerations with faecal contamination (10%, 38% and 42%) were the major factors that led to lung condemnation at ANA, QTA and EBA, respectively. Other causes of lung condemnations were haemorrhage (10%) for QTA and pleurisy (12%) for EBA. The weight loss of lungs during the active abattoir survey was 6450 kg, while the associated monetary loss was estimated as ZAR 29 025 (USD 2706). This study identified major causes of lung condemnation as pleuritis, improper evisceration, pneumonia, abscesses, haemorrhages and lung worms and their associated monetary losses. The results of this study may be useful as baseline data for future comparison in similar surveys, for tracking of some zoonotic diseases affecting lungs and for further research in the Eastern Cape Province or other provinces of South Africa.

## Introduction

Meat is a valuable food commodity in South Africa (Hagen-Zanker, Morgan & Meth 2011). Meat consumption in South Africa is estimated at 41.0 kg per capita per year and is rated the second highest in Africa (Cawthorn, Steinman & Hoffman 2013; FAO 2009). Livestock production aimed at providing meat for the population has seen the South African national cattle herd increase by about 6 million head since the 1970s and it currently numbers about 14 million (Palmer & Ainslie 2006). Over two-thirds of the 14 million cattle in South Africa are found in communal areas (National Department of Agriculture Directorate 2008) with the estimated number of cattle in the Eastern Cape (EC) being over 3.2 million (Tada, Muchenje & Dzama 2013). Livestock production is hindered by various diseases and non-disease factors. According to Fitzpatrick (2013), diseases are the main constraint to efficient livestock production and both endemic and exotic diseases result in mortality and morbidity and hence less food than should ideally be available from current farming systems.

The slaughterhouse is the final destination for most food animals and is the focal point in the farm- to-fork chain for meat products (Dupuy *et al*. 2013). The need for food safety and disease control necessitates meat inspection (MI) at the abattoir. MI involves the screening of animals and meat for fitness for human consumption and is one of the most widely implemented and longest running systems of surveillance (Stärk *et al*. 2014). It can follow traditional or risk-based approaches, and it covers the whole slaughter process that begins with the ante mortem inspection, then stunning and ends at the step where the carcass is placed in the cooler. MI serves to remove gross abnormalities from meat and its products, to prevent the distribution of contaminated meat and to assist in the detection and eradication of certain livestock diseases (Van Llogtestijn 1993) and potentially zoonotic infections.

The proportion of cattle with offal, partial or whole carcass condemnation could be a useful indicator for animal health syndromic surveillance purposes (Dupuy *et al*. 2014) as well as for detecting emerging diseases (Dupuy *et al*. 2013). Data on cattle condemnation provide information on the epidemiology of livestock diseases and give an indication of the extent of public exposure to certain zoonotic diseases. Abattoir condemnations data can also be used to estimate the direct financial losses incurred through condemnation of affected organs and carcasses (Nfi & Alonge 1987; Van Llogtestijn 1993).

In several African countries, there have been reports of substantial wastage of carcasses or offal because of condemnation (Cadmus & Adesokan 2009; Dupuy *et al*. 2014). However, there is a scarcity of information in this regard in South Africa. As South Africa’s population increases, there will be more demand for limited food resources and hence the need to document the various causes of food (meat) wastage. The implications of zoonotic and emerging zoonotic disease further emphasise the vital role of information obtained from MI in the enhancement of public health and food safety. Losses because of condemnation affect the farmer directly and the economy indirectly (Bekele, Tesfaye & Getachew 2010; Ramajo *et al*. 2001). Therefore, this study was aimed at identifying the major causes of lung condemnation and the associated financial implications.

## Materials and methods

### Ethics statement

This research was approved by the University of Fort Hare Research Ethics Committee, and an approval certificate was issued with reference number MUS071SJAJ01. Similarly, before the commencement of the research, a letter was written to the Department of Agriculture Forestry and Fisheries (DAFF) and the participating abattoirs requesting approval of the research. The management of these abattoirs and DAFF issued a letter of permission for the condemnation records to be used for this research.

### Study area and site description

The study was carried out in the EC Province, which is located in the south-eastern region of South Africa. It is bordered on the north by the Free State and Lesotho, KwaZulu-Natal in the north-east, the Indian Ocean to its south and south-eastern borders, and Western and Northern Cape in the west (Bradshaw *et al*. 2003). It is the third largest province in the country stretching over an area of approximately 169 580 km^2^, that is, 13.9% of the total land area of South Africa. It has a high population density estimated at 41 persons per square kilometre. About 63% of the province’s population lives in rural areas (Carabin *et al*. 2006). The EC Province has the highest unemployment rate in the country with the poverty index showing approximately 47% of households living well below the poverty line (Bradshaw *et al*. 2003).

The study was conducted at three abattoirs, which are all located in the EC Province, and were designated ANA, QTA and EBA for ease of reference and confidentiality. The low-throughput abattoir was represented by the acronym (ANA), and located at 32°80’ S 26°90’ E (Chulayo & Muchenje 2013) while the high-throughput abattoirs were QTA and EBA and were located at 31°54’ S 26°53’ E and 32°97’ S 27°87’ E, respectively. The EC Province receives approximately 480 mm of rainfall per year and is situated at an altitude of between 586 m a.s.l. and 2371 m a.s.l. (Mpakama, Chulayo & Muchenje 2014). The temperatures in the EC during the period of the active survey ranged from 18 °C to 25 °C with mean temperatures of 20.5 ºC. The vegetation in the EC ranges from grasslands and thicket to forests and bushveld with *Acacia karroo*, *Themeda triandra* and *Digitaria eriantha* being the most dominant plant species (Muchenje *et al*. 2008). Male and female cattle of different ages and breeds were included in the retrospective and active MI surveys; however, young animals used as veal were not included as these were not expected to manifest diseases leading to meat rejection at the abattoir. Retrospective implied the study of abattoir records from 2010 to 2012 and the active MI referred to the post-slaughter inspection of carcasses from July to December 2013. The study cattle were brought from different locations in the EC Province.

### Sample size determination and sampling

Sample size was calculated based on the formula given by Thrusfield (2005) with 95% confidence interval, 50% expected prevalence and 5% desired absolute precision. The active study sample size was initially 382 carcasses for the high-throughput abattoirs (QTA and EBA) and 176 carcasses for the low-throughput abattoir (ANA). However, the numbers were adjusted or increased to 1146 and 229 carcasses, respectively; this was necessary to increase precision and also to account for a large number of animals slaughtered at the abattoirs during the years in retrospect from which the secondary data were obtained. The number of cattle slaughtered in the three abattoirs varied and the active study sample size was maximised proportionally to ANA (229), QTA (458) and EBA (687).

The sampling procedure was conducted using mixed-method sampling (Teddlie & Yu 2007), which involved a typical case sampling technique of purposeful sampling for ANA, while systematic random sampling was used for QTA and EBA (Thrusfield 2005). Sampling units for ANA involved the selection of rejected offal and recording cases as per unit, while sampling units for QTA and EBA were selected at equal intervals with the first animal being selected randomly. The total number of animals slaughtered during the preceding year (2012) was obtained from the abattoir records as 26 401 cattle corresponding to 520, 4078 and 21 803 for ANA, QTA and EBA, respectively. The active MI was carried out for 6 months in 2013 and during this time, the number of slaughtered animals at the three abattoirs was 20 791 cattle, corresponding to 322, 3788 and 16 681 for ANA, QTA and EBA abattoirs, respectively. Sampling interval was thus computed as the total number of animals slaughtered during the study period divided by the required sample size (Regassa *et al*. 2013). Therefore, the sampling interval for the QTA was 8 (3785/458) and for EBA 24 (16 681/687), with the first bovine being chosen randomly from the first 8 and 24 animals, respectively. Subsequently, every 8th and 24th bovine was included in the samples during the slaughter operation.

### Study types

A cross-sectional study was employed using retrospective abattoir records from 2010 to 2012 as well as an active abattoir survey from July to December 2013. Information regarding the slaughter of 51 302 cattle from abattoir records and 1374 cattle from the active abattoir survey was analysed. The abattoir survey, which involved MI, was conducted by authorised meat inspectors at the various abattoirs. Causes of condemnation of lungs were recorded on spreadsheets to evaluate the economic implication of such condemnation. Active MI was carried out or performed by the procedure specified by the *South African Meat Safety Act* (Act No. 40 of 2000), using palpation of organs and visual inspection, with inspectors incising at various suspicious and diseased focal points to determine the health status of the meat or offal. Condemned organs upon slaughter were passed through a condemnation chamber to the condemnation room where they were treated and disposed off.

The carcasses that were included in the study were thoroughly screened after slaughter for the presence of lesions. Organs with gross pathology were separated, classified and recorded in line with the guidelines for MI in developing countries (Herenda *et al*. 1994). The lungs that were classified as totally condemned were rejected and destroyed to avoid human consumption. In cases of partial condemnation, trimmed offal weight was estimated based on kilogram weight of daily total trimmed organs. Trimmed lungs were collected into a condemnation drum and measured or weighed using a digital measuring scale (Ansutek M1/M2 Portable Crane Scale, Ansutek Commercial Ltd, New Zealand) with the weight of the condemnation drum subtracted to get the accurate kilogram weight of condemned organ or organs. The record of partial condemnation was taken on the days the abattoirs were visited during the 6 months of the research, while the monetary loss was calculated based on the market prices per kilogram of lung during the study.

### Direct financial loss

Monetary losses were calculated based on the then current market price (Borji, Azizzadeh & Kamelli 2012; Regassa *et al*. 2013; Terefe, Wondimu & Gachen 2012; Zewdu, Yechale & Makwoya 2010) of whole organ or lung in South African Rand (ZAR). The prices for trimmed organ and carcass were estimated based on the price per kilogram of condemned organ or carcass using the ZAR. The prevailing exchange rate at the time the study was conducted was 1.00 USD – 10.7257 ZAR or 1.00 ZAR = 0.0932637 USD. The average weight of 10 lungs was taken and the measurement recorded as 7.8 kg. Lung prices were obtained from the three abattoirs, and the average price in Rand was ZAR 38 (USD 3.5). Financial loss associated with lung condemnation was in the form of losses because of whole or partial lung rejection. For partial condemnations, diseased areas of lung were cut off and condemned. Otherwise, the whole organ was condemned, in which case losses were according to the size or weight of the condemned organ. Abattoirs bore the losses regarding partial condemnation, while losses accruing from condemnations of whole organs and carcass were referred to the farmers.

### Data analysis

Data extracted from retrospective records and active abattoir MIs were entered into Microsoft Excel (MS Excel 2007) spreadsheets. Carcass and organ condemnation rates were determined using simple descriptive statistics of statistical analysis system SAS (2003) and compared using chi-square test at a critical probability of *p* < 0.05. The variables compared included proportions of lung lesions by years and abattoirs. Condemnation rate for retrospective data was defined as the proportion of lungs condemned to the total number of lungs slaughtered in a year. Data were then analysed on a year-to-year basis. The rate of condemnation for active MI was calculated as the proportion of lungs condemned to the total number of lungs examined during the study period (July – December 2013) (Regassa *et al*. 2013). Monetary loss was calculated using current abattoir market prices of lungs and carcass (loss in kg).

## Results

**FIGURE 1:**
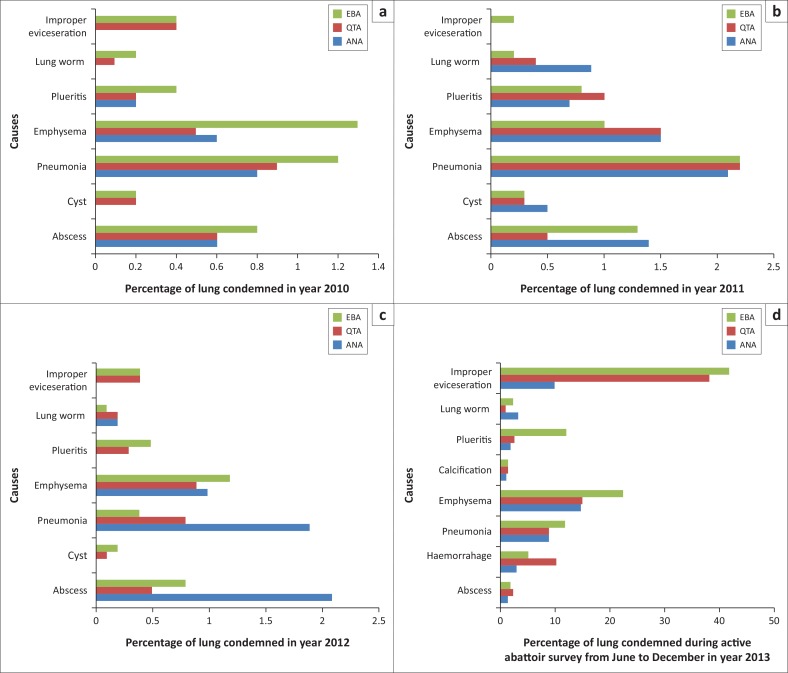
Causes (%) of cattle lung condemnations in the Eastern Cape Province South Africa. For the year (a) 2010, (b) 2011, (c) 2012 and (d) 2013.

A total of 51 302 cattle were slaughtered from 2010 to 2012 and 17 480 kg of lungs were lost because of condemnation during this period (Table 1). A total number of 1374 cattle were slaughtered during the active MI and 6450 kg of lungs were lost during this period (July – December 2013) (Table 3). The percentage of lungs condemned in 2010, 2011 and 2012 were 4.04%, 6.03% and 3.58%, respectively (Table 2). There was a significant difference (*p* < 0.05) in the number of condemned lungs at each abattoir. Monetary losses associated with the condemnation of lungs were ZAR 15 770 for 2010, ZAR 33 516 for 2011 and ZAR 35 872 for 2012 based on the average price per kilogram of lung tissue during those years. The amounts in USD were 1470, 3125 and 3344 for the years 2010, 2011 and 2012, respectively (Table 1). The leading causes of lung condemnation in 2010 were emphysema (1.12%), closely followed by pneumonia (1.09%) and the lowest number of lung condemnation was because of cysticercosis (0.17%). Conversely, in 2011, pneumonia (2.21%), emphysema (1.14%) and improper evisceration (0.13%) accounted for the most lung condemnation. In 2012, the top causes were emphysema (1.16%) and abscessation (0.77%) and lung worm (0.14%) caused the least lung condemnations (Table 2 and Figure 1). During the active abattoir survey, the major cause of lung condemnations was emphysema, while improper evisceration led to the condemnation of 2703 kg of lungs (Table 3 and Figure 1).

**TABLE 1:** Losses associated with lung condemnation in three abattoirs from 2010 to 2012.

Abattoir	2010 (*n* = 10 276)				2011 (*n* = 4625)				2012 (*n* = 6401)			
	Nolc	Wt loss (kg)	Loss (ZAR)	Loss (USD)	Nolc	Wt loss (kg)	Loss (ZAR)	Loss (USD)	Nolc	Wt loss (kg)	Loss (ZAR)	Loss (USD)
ANA	11	86	418	39	31	242	1178	110	27	211	1026	96
QTA	63	491	2394	223	261	2036	9918	925	129	1006	4902	457
EBA	341	2660	12 958	1208	590	4602	22 420	2090	788	6146	29 944	2792
**Total**	**415**	**3237**	**15 770**	**1470**	**882**	**6880**	**33 516**	**3125**	**944**	**7363**	**35 872**	**3344**

ANA, low-throughput abattoir; QTA and EBA, high-throughput abattoir; Nolc, number of lungs condemned; Wt loss, weight loss; ZAR, South African rand; USD indicates United State dollar.

## Discussion

Abattoir MI and slaughter records contribute to disease surveillance and control through macroscopic identification and recording of basic lesions, thus a first step in monitoring diseases in the national herd and flock. The information received through surveillance studies provides feedback to the veterinary service for the control and eradication of animal diseases and the protection of public from zoonotic hazards (Regassa *et al*. 2013; Van Llogtestijn 1993). It also reveals causes of meat condemnation even in apparently healthy animals, and this information can be communicated back to the farmer in order to improve farm management and husbandry.

An estimated 17479.8 kg of the lung were lost in the period covered for the retrospective study, while lungs weighing 6450 kg were lost during the active abattoir study. Factors responsible for the loss included: abscessation, tapeworm cysts, pneumonia, emphysema, pleuritis, lung worm, improper evisceration and calcification. Our results indicate that pneumonia, emphysema and abscessation were the leading causes of lung condemnation. These findings (Table 2) were similar to those of other researchers (Fekadu, Legesse & Tesfaye 2012; Lat-Lat, Sani & Sheikh-Omar 2006; Phiri 2006), who reported similar lesions leading to losses of 0.5%, 0.8% and 1.11%, respectively. Comparatively, much higher loss of 8.2% cattle lungs was recorded by Mellau, Nonga and Karimuribo (2010b).

**TABLE 2:** Causes of lung condemnation in cattle slaughtered at three abattoirs from 2010 to 2012 in the Eastern Cape Province South Africa.

Organ affected	Causes of condemnation	Number of condemned organs (condemnation rate) during the 3 years														
		2010 (*n* = 10 276)					2011 (*n* = 14 625)					2012 (*n* = 26 401)				
		(*n* = 502) ANA	(*n* = 2127) QTA	(*n* = 7647) EBA	Total		(*n* = 437) ANA	(*n* = 4414) QTA	(*n* = 9774) EBA	Total		(*n* = 520) ANA	(*n* = 4078) QTA	(*n* = 21 803) EBA	Total	
					*n*	%				*n*	%				*n*	%
Lung	Abscessation	3	12	58	73	0.71	6	23	126	155	1.06	11	20	172	203	0.77
	Cysticercosis/cyst	0	5	12	17	0.17	2	15	29	46	0.31	0	4	41	45	0.17
	Pneumonia	4	20	88	112	1.09	9	96	218	323	2.21	10	31	88	129	0.49
	Emphysema	3	11	101	115	1.12	7	66	94	167	1.14	5	38	263	306	1.16
	Pleuritis	1	5	34	40	0.39	3	45	83	131	0.9	0	11	109	120	0.45
	Lung worm	0	2	16	18	0.18	4	16	21	41	0.28	1	8	29	38	0.14
	Imp Env	0	8	32	40	0.39	0	0	19	19	0.13	0	17	86	103	0.39
**Total**	**-**	**11**	**63**	**341**	**415**	**4.04**	**31**	**261**	**590**	**882**	**6.03**	**27**	**129**	**788**	**944**	**3.58**

ZAR, South African rand; Imp. Env., improper evisceration; ANA, low-throughput abattoir; QTA and EBA, high-throughput abattoir.

In the present study, lung abscessation was responsible for 2%, 3% and 2% of lung loss in the three abattoirs studied ANA, QTA and EBA, respectively (Table 3). Lung abscessation reported in this study was also similar to the findings by Lat-Lat *et al*. (2006) in peninsular Malaysia, Phiri (2006) in western Zambia, Fekadu *et al*. (2012) in southwestern Ethiopia and Alembrhan and Haylegebriel (2013) in northern Ethiopia. However, a higher rate of lung condemnation because of abscesses were report by Cadmus and Adesokan (2009) in western Nigeria, Denbarga, Demewez and Sherefaw (2011) in northern Ethiopia and Dupuy *et al*. (2014) in France. Accumulation of pus (abscessation) in the lungs indicates the presence of pyogenic bacteria and most often is because of secondary bacterial infection. Lung abscessation may originate from infected emboli in the blood arising from other septic organs, or areas as in the case of endocarditis, lymphadenitis, mastitis and metritis. It was documented in Tanzania that *Mannheimia haemolytica* (formerly *Pasteurella haemolytica*) and *Trueperella pyogenes* (formerly *Actinomyces pyogenes*) are the main causes of lung abscessation in cattle (Mellau *et al*. 2010b).

**TABLE 3:** Conditions that led to the condemnation of lung and associated monetary loss (in ZAR) during the active abattoir survey (July–December 2013).

Causes of condemnation	Number (%) of condemned lung (*n* = 229) (*n* = 458) (*n* = 687) during active abattoir meat inspection											
	Number affected (Loss in %)						Loss in kg			Monetary loss		
	ANA		QTA		EBA		ANA	QTA	EBA	kg	ZAR	USD
	*n*	%	*n*	%	*n*	%						
Abscess	4	2	12	3	13	2	23.2	63.6	81.4	168.2	757	71
Haemorrhage	7	3	48	10	36	5	41.6	254.4	170.8	466.8	2101	196
Pneumonia	21	9	41	9	82	12	113.8	229.8	429.6	773.2	3479	324
Emphysema	34	15	69	15	155	23	225.2	418.2	909.0	1552.4	6986	651
Calcification	3	1	7	2	11	2	13.4	24.6	75.8	113.8	512	48
Pleuritis	5	2	13	3	84	12	19.0	80.4	445.2	544.6	2451	229
Lung worm	8	3	5	1	17	2	32.4	27.0	68.6	128.0	576	54
Imp Env	23	10	175	38	287	42	99.4	1165.0	1438.6	2703.0	12 164	1134
**Total**	**105**	**46**	**370**	**81**	**685**	**100**	**568.0**	**2263.0**	**3619.0**	**6450.0**	**29 025**	**2706**

Imp. Env., improper evisceration, ZAR, South African rand, ANA, low-throughput abattoir, QTA and EBA, high-throughput abattoir.

Information extracted from abattoir records indicated 0.17%, 0.31% and 0.17% prevalence’s of cysticercosis cysts for the years 2010, 2011 and 2012. Several studies (Carabin *et al*. 2006; Mafojane *et al*. 2003; Torgerson & Macpherson 2011) in eastern and southern South Africa, EC Province and globally, respectively, referred to the possibility of finding hydatidosis and cysticercosis in lungs. Reports by Phiri (2006) in Zambia, Cadmus and Adesokan (2009) in western Nigeria and Tolosa *et al*. (2009) and Megersa *et al*. (2010) in Ethiopia, revealed a high prevalence of cysticercosis in different parts of Africa. While others (Ansari-Lari 2005; Damet & Egene 2013; Debas & Ibrahim 2013; Kambarage *et al*. 1995; Kebede *et al*. 2009; Mellau, Nonga & Karimuribo 2011; Tadesse *et al*. 2014) in Tanzania, Iran and Ethiopia observed various prevalences of hydatid cysts in ruminants. The active abattoir survey did not detect any viable cysts. However, mineralised foci in different lobes of the lungs were suspected to be dead cysts of *Echinococcus granulosus* and *Taenia saginata*. No accurate data on the prevalence of bovine cysticercosis in the EC Province have been reported. This may be partly because of poor reliability of the available diagnostic tests and the high costs of performing these tests under field conditions. Most of the prevalence studies have relied on slaughter data (Njoroge *et al*. 2002) in Kenya. According to Kebede *et al*. (2009) in Ethiopia, the lungs are predominantly affected by hydatidosis possibly because of the presence of larger capillaries, which aid the migrating hexacanth embryo (*Echinococcus* oncosphere). These embryos adopt the portal vein route and primarily negotiate the hepatic and pulmonary filtering systems sequentially before reaching any other peripheral organ (Getaw *et al*. 2010).

Moreover, the practice by some farmers in the EC Province of allowing dogs to roam with livestock likely aids in disseminating infestation with *E. granulosus*, as it facilitates the interface between dogs and domestic livestock. Dogs are the definitive host for *E. granulosus*, and the potential for environmental contamination is high under these circumstances with the parasite easily transmitted to ruminants and humans (Ernest *et al*. 2009). The non-viability of the cysts obtained in the present study may be because of prior medication or the animals’ immune system defences against invading parasites. This viewpoint was supported by the findings of Abunna *et al*. (2008) during an investigation of bovine cysticercosis at Awassa municipal abattoir in Ethiopia. The differences between the incidences found in the current study and those reported by others in Ethiopia, Kenya and Tanzania (Abunna *et al*. 2012; Debas & Ibrahim 2013; Getaw *et al*. 2010; Kebede *et al*. 2009; Mellau *et al*. 2011; Njoroge *et al*. 2002; Tadesse *et al*. 2014) may be ascribed to the differences in the agro-climatic conditions of the study areas, environmental conditions that are conducive for parasitic replication and availability of a definitive host (Mellau *et al*. 2011). Furthermore, other factors that could account for the current result is grazing patterns of livestock and the nature of the pasture, the human culture of eating raw or undercooked meat, the likelihood of the same or sufficient diagnostic incisions being made at the inspection site from one abattoir to the other (Kambarage *et al*. 1995) and also the dose and viability of eggs and or larvae consumed (Tadesse *et al*. 2014).

The high rate of cysticercosis in developing countries has often been associated with poor sanitary infrastructure, inadequate public awareness of the condition and improper disposal of sewage (Tolosa *et al*. 2009). These conditions aptly depict the sanitary situation in the rural locations of the EC Province and may be responsible for the prevalence seen in cattle brought for slaughter especially by rural farmers. The method of MI, the expertise and precision of the meat inspector (Cadmus & Adesokan 2009; Lat-Lat *et al*. 2006; Mellau, Nonga & Karimuribo 2010a), the differences in farm management, as well as the sampling method, lesion location and the stage of degeneration of the cysticercoids (Hill *et al*. 2014; Kebede 2008), plus other factors can significantly contribute to the variations of recorded prevalence of bovine cysticercosis. The prevalence obtained in the current study may be an underestimation of the true prevalence because of improper recording and an undefined number of lungs condemned for abscessation.

Pneumonia was a leading cause of lung condemnation (9%, 9%, and 12%) in this study. In ruminants, pneumonia is a complex condition that involves the interaction between the animal’s immunity, physiological system, multiple pathogenic agents such as bacteria, virus and mycoplasmas and also environmental factors (Caswell 2014; Grubor *et al*. 2004; Katsuda *et al*. 2009; Mellau *et al*. 2010a; Meyerholz & Ackermann 2005; Singh, Ritchey & Confer 2011; Storz 1985). Cold and dry conditions such as prevail in winter may predispose cattle to pneumonia. Furthermore, Mellau *et al*. (2011), reported that poor housing and overcrowding that may subject the animals to various stresses like cold, wind, rain and dust may result in opportunistic bacterial infection by organisms like *Pasteurella multocida*, *M. haemolytica* and *T. pyogenes*. Other stress-related factors responsible for pneumonia are penetration of lungs by foreign bodies (Fekadu *et al*. 2012), parasitism such as massive infestations of the respiratory tract with ascarid larvae and lung worm (Kambarage *et al*. 1995) and fungus-contaminated feed supplied to the animals during transportation or at cattle sales markets. Exposure to environmental dust or fatigue during a long journey in search of pastures as occurs in Nigeria can also predispose animals to pneumonia (Cadmus & Adesokan 2009). Cattle raised under extensive grazing systems as is popular in the EC Province, similarly walk long distances in search of pastures, likely predisposing livestock to pneumonia and thereby contributing to the numerous cases of pneumonia observed in this study. Seasonal changes in weather conditions especially from autumn to winter and from winter to spring are associated with wind and dust. This condition predisposes cattle to several respiratory diseases and may contribute to variations in the number of lungs rejected because of pneumonia as reported in Tanzania and Ethiopia (Mellau *et al*. 2010b; Regassa *et al*. 2013).

Pulmonary emphysema (15%, 15% and 23%) was recorded as the second leading cause of lung condemnation. Emphysema in cattle is typically secondary to or associated with some primary respiratory disease conditions such as infectious bovine rhinotracheitis, pneumonic pasteurellosis, malignant catarrhal fever, mycoplasmal infection, leptospirosis and some cases of septicaemia and endocarditis, as reported in Tanziania (Mellau *et al*. 2010b). Because of well-developed pulmonary interlobular septae and lack of collateral ventilation in sheep, pigs, and particularly in cattle, these species are susceptible to interstitial emphysema (Mellau *et al*. 2010b). However, some cases of emphysema have been recorded in slaughter animals because of extensive agonal phase gasping during slaughter especially when animals are slaughtered without captive bolt stunning (Mellau *et al*. 2010b). Some cattle both at ANA and QTA were shot with bullets to the head and bled out on the farms because of the owners’ inability to restrain them for onward live transportation to the abattoir. Others at ANA were shot and bled out while at the abattoir because these animals were aggressive upon arrival and could not be herded into the stunning box. Situations such as this may have caused agonal emphysema and consequently increased the percentage of lungs discarded.

The age of slaughter animals has also been associated with emphysema, with very old cows reportedly more prone to emphysema when slaughtered (Mellau *et al*. 2010b) than young ones. Hypersensitivity pneumonia, also called ‘farmer’s lung’ as reported in France, affects mainly adult dairy cattle after exposure to hay with high moisture content and has been associated with emphysema (Dupuy *et al*. 2013). Stress factors including fatigue during long journeys in search of pastures and exposure to polluted air in their environments, predispose animals to respiratory distress and have been shown to promote lung emphysema (Regassa *et al*. 2013).

The percentages of worms in the lungs observed in this study (3%, 1% and 2%) were similar to those found by Lat-Lat *et al*. (2006) in cattle and buffalo in Malaysia and by Regassa *et al*. (2013) in sheep and goats in Ethiopia, but it was higher in an earlier Ethiopian study by Regassa *et al*. (2010). Lung worms have been reported to occur sporadically in the highlands of tropical and sub-tropical regions similar to some South African regions and to be common and widespread in cattle of temperate regions around the world (Lat-Lat *et al*. 2006). The slaughter records of the current study showed a higher prevalence of lung worms in summer than winter in the EC Province. The irritation associated with the feeding activities of the adult worms in the respiratory tract predisposes the animals to bronchitis, allergic reactions, pneumonia with massive infestations and calcified cysts. These lesions are associated with *Dictyocaulus viviparus*, *Dictyocaulus filaria* and *Muellerius capillaris* (Lat-Lat *et al*. 2006; Mellau *et al*. 2010; Regassa *et al*. 2013).

In generally, helminth infestation can result in losses in productivity through reduction in feed intake and feed conversion efficiency, loss of blood (associated with some intestinal helminths) and even death. Farmers in rural areas lack access to adequate veterinary extension services and information regarding control and treatment of animal helminthosis. According to Tsotetsi and Mbati (2003), cattle on communal grazing play a major role in the socio-economics of small-scale farmers living in villages and townships in South Africa, yet these cattle are seldom treated for internal parasites. This likely explains the numbers of lungs condemned because of lung worm at ANA (3%) when compared to the number of animals examined during the study and in comparison to those condemned at QTA (1%) and EBA (2%).

Pleuritis (pleurisy) is inflammation of the pleural membrane that surrounds and protects the lungs, and healing may result in chronic fibrous adhesions between the lungs and chest wall (Branch & Club 2011). In adult cattle, these may sometimes be a legacy of respiratory disease contracted as intensively reared calves (Edwards, Johnston & Mead 1997). The percentages of lungs condemned because of pleuritis in the present study (2%, 3% and 12%) were similar to results obtained by Lat-Lat *et al*. (2006) and Regassa *et al*. (2013) in Malaysian and Ethiopian ruminants but was lower than results obtained by Kambarage *et al*. (1995) in Tanzania. This may be because of differences in geographical locations, aetiologies, different definitions used (Mellau *et al*. 2010b) or to differences in personal assessments, season and definitive diagnosis of pleurisy by the different studies.

Improper evisceration accounted for partial condemnation of 2703 kg weight of lung, and thus economic loss to the abattoir. Improper evisceration, which usually leads to faecal contamination of meat or offals, has been attributed to a failure by slaughter personal to carefully remove the pluck and alimentary canal during the slaughtering process (Dupuy *et al*. 2013). This compromises meat hygiene and quality by contaminating carcasses or organs with potentially highly pathogenic organisms like *Salmonella* and verotoxin-producing *Escherichia coli* that are found in the alimentary canal of animals (Blagojevic & Antic 2014). Our percentages of 10%, 38% and 42% condemnations of lungs because of faecal contamination were higher than the finding of Regassa *et al*. (2013), who reported a 4.8% loss of lungs because of faulty evisceration in ruminants in Ethiopia. In our study, evisceration problems were probably because of unskilled and inappropriately trained workers. In addition, inadequate numbers of workers at the eviscerating unit may lead to workers rushing to meet daily slaughter targets, thus leading to improper evisceration and lung faecal contamination.

### Financial implications of carcass or organ condemnations in the three abattoirs

Wholesale prices during the years of the study were used for the estimation of the financial loss associated with condemnation. Thus, it is very likely that higher monetary loss is possible if retail market prices were used. Furthermore, condemnations were made by meat inspectors, and the likelihood of human error as found in other studies may have led to some lungs being overlooked. Some of these errors include improper identification of lesions, improper recording, lack of expertise and precision of meat inspectors and poor sampling method (Cadmus & Adesokan 2009; Lat-Lat *et al*. 2006; Mellau *et al*. 2011). In our study, 17 480 kg of lung from 2010 to 2012 were lost and 6450 kg was lost during the active MI in 2013. At ANA, 3237 kg was lost, while at QTA and EBA, 6880 and 7363 kg were lost, respectively. The differences noted between the abattoirs may have been because of differences in slaughter capacity and meat inspector efficiency. Monetary loss estimated from 2010 to 2012 was 22 239 ZAR (USD 7939), while condemned lung loss during the active abattoir survey was worth ZAR 29 025 (USD 2706).

Current information on the quantity and financial implications of meat condemnation in the EC Province is scarce or unavailable. Elsewhere in Africa, studies of this nature have shown various degrees of financial losses (Denbarga *et al*. 2011; Fekadu *et al*. 2012; Kithuka *et al*. 2002; Mwabonimana *et al*. 2010). The majority of condemned lung losses observed during the active EC Province abattoir survey were because of improper evisceration, emphysema, pneumonia, pleuritis and haemorrhages. Several studies (Mellau *et al*. 2010b, 2011; Phiri 2006; Regassa *et al*. 2013; Tadesse *et al*. 2014; Torgerson & Macpherson 2011) have indicated that disease and non-disease factors lead to lung condemnation, which results in financial loss, the burden of which directly and indirectly affects the farmer, the abattoir and the economy of South Africa.

## Conclusion

Bovine cysticercosis, pleuritis, calcification, faecal contamination from improper evisceration, pneumonia, abscessation, haemorrhages and lung worms were major causes of lung condemnation in this study, resulting in a considerable direct financial loss to the farmer and abattoir. An efficient MI service is crucial in basic monitoring of animal diseases with substantial economic and public health significance. Secondary MI by veterinarians with correct sampling of lesions for specific aetiological diagnoses by supporting diagnostic laboratories, although costly, would greatly increase the amount of important information obtainable from abattoirs and contribute to combating controllable and notifiable diseases as well as zoonotic diseases. For example, the use of histopathology, immunohistochemistry, parasite identification, bacterial culture, antibiograms to follow antibiotic sensitivity of bacteria, trends in anthemintic resistance and molecular biological techniques for parasites, viruses, bacteria and fungi detection would greatly improve MI and disease surveillance in an abattoir. Public awareness campaigns are recommended regarding the public health implications of hydatidosis and cysticercosis, and efforts by relevant government agencies are needed to assist farmers in early diagnosis, prevention and control of the diseases. Ongoing farmer education through a vibrant veterinary extension service is central to understanding the seasonal trends in disease and effective prophylactic or treatment regimens needed to break disease cycles. Good slaughtering and evisceration practices need to be implemented at abattoirs to reduce lung condemnation because of improper bleeding and contamination.
